# Coexistence of Moderate-to-Severe Obstructive Sleep Apnea and Inflammation Accelerates the Risk of Progression of Arterial Stiffness: A Prospective 6-Year Study

**DOI:** 10.3390/life12111823

**Published:** 2022-11-08

**Authors:** Jinkwan Kim, Dae Wui Yoon, Sungmin Myoung, Seung Ku Lee, Chol Shin

**Affiliations:** 1 Department of Biomedical Laboratory Science, College of Health Science, Jungwon University, Geo-San 28024, Korea; 2 Department of Medical Information and Administration, College of Health Science, Jungwon University, Geo-San 28024, Korea; 3 Institute of Human Genomic Study, Korea University Ansan Hospital, Korea University, Ansan 15355, Korea; 4 Department of Pulmonary Sleep and Critical Care Medicine Disorder Center, College of Medicine, Korea University, Ansan 15355, Korea

**Keywords:** obstructive sleep apnea, inflammation, high sensitivity C-reactive protein, arterial stiffness, pulse wave velocity, cardiovascular disease

## Abstract

Both obstructive sleep apnea (OSA) and inflammation have now been recognized as imposing substantial cardiometabolic risk. However, no prospective study has reported whether the coexistence of OSA and inflammation exacerbates the progressive arterial stiffening. Thus, the purpose of this study is to examine whether these conditions increase the risk of the progression of arterial stiffening. A total of 1945 participants were randomly selected for the study. Subjects with elevated inflammation were divided by high-sensitivity C-reactive protein (hsCRP) levels. A polysomnography and brachial–ankle pulse wave velocity (baPWV) were performed. The elevation of the baPWV was defined as the levels in the highest quartile of the baPWV. The percentage of the elevated baPWV and the change in the baPWV (ΔbaPWV) were higher in individuals with OSA and higher hsCRP levels. After adjusting for confounders, the participants with OSA and inflammation in the groups not treated with antihypertensive medication had a higher risk of an elevated ΔbaPWV in contrast to those with neither variable. Particularly, the alteration in the baPWV differed significantly based on the existence of moderate-to-severe OSA and inflammation at the 6-year follow-up. In combination, these conditions are associated with an accelerated risk of a future burden of the progression of the arterial stiffness, suggesting a potential important role in the increased risk of CVD.

## 1. Introduction

Obstructive sleep apnea (OSA) is recognized as a common frequent condition and is associated with increased cardiometabolic risks [1,2,3]. The high-sensitivity C-reactive protein (hsCRP), synthesized by the liver, is a biomarker of the underlying systemic inflammation and represents a crucial marker in CVD [4,5]. Not surprisingly, accumulating evidence has reported that the elevation of inflammatory markers such as the hsCRP, interleukin (IL)-6, and tumor necrosis factor (TNF)-α were shown in subjects with OSA, and these were significantly associated with increased excessive daytime sleepiness. Nevertheless, not all OSA subjects showed increased inflammatory markers [6,7,8], proposing that an interplay between genetic variability and various factors, such as obesity and race, might represent a crucial confounding factor of the inflammatory phenotype observed in OSA.

Over the last two decades, substantial data from clinical and epidemiological studies have indicated that arterial stiffening is implicated in various cardiometabolic components [9,10]. The brachial–ankle pulse wave velocity (baPWV), which is regarded as a subclinical biomarker of cardiometabolic disease, has been applied in various settings, owing to its simplicity [11]. The evidence from clinical or epidemiological studies has shown that the PWV levels in subjects with OSA remarkably increase and significantly reduce after treatment with continuous positive airway pressure (CPAP) [12,13,14]. Moreover, a recent study showed that the alteration in the baPWV over 6 years was remarkably different in regard to the OSA severity independently of the obesity status in subjects without hypertension, suggesting that moderate-to-severe OSA is a potential predictor of progressive arterial stiffening [15]. However, no study has been reported about whether the coexistence of OSA and inflammation accelerates the risk of progressive arterial stiffening in a cohort study. Herein, the purpose of this study is to investigate whether the coexistence of OSA and inflammation exacerbates the risk of progressive arterial stiffening in a prospective setting.

## 2. Materials and Methods

### 2.1. Subjects

The Korean Genome and Epidemiology Study (KoGES) was initiated in 2001 to identify the risk factors and to examine the prevalence of chronic disease among Korean people aged 40–69 years. Detailed information on the study design and purposes of the KoGES can be found in previous studies [16,17,18,19,20]. The study protocol was approved by the Human Subjects Review Committee at Korea University Ansan Hospital (Protocol ID: ED0624). All procedures were carried out in agreement with the pertinent protocols [20]. We used the 4th examination data acquired between 2007 and 2009 and the 7th examination data between 2013 and 2015 for follow-up. Nocturnal unattended polysomnographic data were acquired between September 2009 and March 2012. We included a total of 1945 participants (1014 men and 931 women) for the study, after excluding cases with insufficient information and extremely skewed biochemical data. In addition, cases with missing or extremely outlier data for baPWV (*n* = 19) and biochemical parameters (*n* = 91) or that had undergone any OSA treatment (*n* = 28) were not included. Eight participants were receiving OSA treatments at the 6-year follow-up period. Finally, we conducted a prospective study including 1945 participants derived from the KoGES. For this cohort, subjects were classified into four groups in relation to the existence of OSA and level of hsCRP. The patient group treated with antihypertensive medication consisted of any participant taking any form of hypertensive medication at baseline.

### 2.2. Overnight Polysomnography

PSG data were acquired using a portable device (Embletta^®^ X-100; Embla Systems, San Carlos, CA, USA) at each participant’s home according to our previous studies [15,17]. Obstructive apnea was defined as a clear decrease (≥90%) from baseline in the amplitude of the nasal pressure with ongoing chest and abdominal movement. Hypopneas were identified if there was a ≥30% reduction in the oronasal flow from baseline, related with at least 4% oxygen desaturation on pulse oximetry. We judged that OSA exists if the AHI score was greater than 5. An AHI > 5 to AHI < 15 and AHI ≥ 15 were classified as mild and moderate-to-severe OSA (MSOSA), respectively. Arousals were scored in agreement with the standard protocol [21,22].

### 2.3. Demographic Profiles of Subjects

All the study subjects were examined about demographic profiles, including health history and sleep problems. The Epworth Sleepiness Scale (ESS) was used for the assessment of daytime sleepiness. Blood glucose, lipid profiles, and hsCRP levels were determined (ADVIA 1650 and 1800, Siemens, Tarrytown, NY, USA) in overnight fasting blood. A high hsCRP level was defined as greater than 1.43 mg/dL for males and greater than 1.15 mg/dL for females, in accordance with the 75th percentile among the entire cohort. Multi-frequency bioelectrical impedance analysis (InBody 720; Biospace, Seoul, Korea) was applied to measure body composition [17,23,24].

### 2.4. Measurement of Arterial Stiffness and Definition of Hypertension and Diabetes Mellitus

BP was measured at baseline and at biennial follow-up visits by trained examiners according to standardized methods [16]. BaPWV was determined to evaluate the arterial stiffening using an automated waveform analyzer (VP1000; Omron Co., Kyoto, Japan) at baseline and repeated at the 4- and 6-year follow-up visits [25]. We defined hypertension (HTN) if systolic BP value was ≥40 mmHg and diastolic BP value was ≥90 mmHg or if antihypertensive medication was used. Diabetes mellitus (DM) was also defined when fasting glucose level was ≥126 mg/dl or taking anti-diabetic medications. BaPWV was determined to be elevated when the cut-off at the highest quartile level was greater than 14.8 m/s for the entire cohort. In addition, ΔbaPWV and elevated ΔbaPWV were determined as the change in baPWV at the 6-year follow-up and as higher than the cut-off at the highest quartile level of ΔbaPWV was greater than 1.9 m/s, respectively.

### 2.5. Statistical Analyses

To examine statistical differences, four groups were determined by one-way analysis of variance (ANOVA) using the Scheffe post hoc test for continuous variables and the chi-square test for categorical variables. We performed a multivariate logistic regression analysis. The adjusting factors for elevation of baPWV or ΔbaPWV in entire cohort were as follows: age, sex, smoking, alcohol status, mean arterial pressure (MAP), fasting glucose, HDL cholesterol, medication for DM and hyperlipidemia, and each of obesity-associated variables (BMI (Model 1), WHR (Model 2), and FM (Model 3)) at baseline and the alteration in each of obesity-related variables (ΔBMI (Model 1), ΔWHR (Model 2), and ΔFM (Model 3)) at the 6-year follow-up. Adjusted odds ratios (ORs) were reported with 95% CIs, referencing that of subjects with neither OSA nor high hsCRP levels. Because HTN medication was expected to contribute to alter baPWV as a major confounding factor [26,27], similar statistical tests were performed to assess ORs for elevation of baPWV or ΔbaPWV in groups divided by the presence or absence of HTN medication. Furthermore, we performed linear mixed model analyses to examine the mean change in baPWV over time in terms of the existence of moderate-to-severe OSA and high hsCRP levels, after controlling for the same variables in Model 1 at baseline and the alteration in BMI at the 6-year follow-up. A two-sided *p* < 0.05 was considered statistically significant. All statistical analyses were performed using SPSS software (version 23.0, IBM Corp., Armonk, NY, USA).

## 3. Results

### 3.1. Study Population

Of the 1945 subjects at baseline study, 284 participants dropped out over the 6-year follow-up period (non-response rate, 14.6%). At the baseline study, 52.1% of the participants were male. The general characteristics of the subjects are presented in Table 1. The subjects were classified into four groups in relation to the presence of OSA and high hsCRP levels. The AHI and the lowest desaturation level (SaO_2_ Nadir) showed a significant difference between the groups (*p* < 0.01). The metabolic profile, including the glucose, high-density lipoprotein (HDL) cholesterol, and triglyceride levels, significantly differed among the four groups (*p* < 0.01). In addition, the percentage of hypertension and diabetes mellitus among the four groups was a statistically significant difference.

### 3.2. The Estimated Risk of Elevated baPWV Based on the Existence of OSA and High hsCRP in Participants at Baseline

The data for the baPWV among the four groups at baseline are shown in Table 2. The mean baPWV in the four groups among the participants not treated with antihypertensive medication (OSA[−]/HsCRP[−] vs. OSA[−]/HsCRP[+] vs. OSA[+]/HsCRP[−] vs. OSA[+]/HsCRP[+], 13.1 ± 1.8 vs. 13.6 ± 2.1 vs. 13.8 ± 1.9 vs. 14.1 ± 1.9 m/s, *p* < 0.01) were significantly different but not in those treated with antihypertensive medication. To examine the odds ratios for the likelihood of the elevated baPWV based on the existence of high hsCRP levels among the non-OSA and OSA participants, univariate and multiple logistic regression analyses were applied in the entire cohort and subgroups with or without antihypertensive medication. After adjustment for the potential confounding factors, including the obesity-associated variables (BMI and waist-to-hip ratio (WHR), and fat mass (FM)/body weight) in the multivariate analyses, we found that the OSA participants with a high hsCRP level in the entire population had a 2.05-fold (Model 1, 95% confidence interval (CI), 1.39, 3.01, *p* < 0.01), 1.67-fold (Model 2, 95% CI, 1.15, 2.43, *p* < 0.01), and 1.84-fold (Model 3, 95% CI, 1.26, 2.69, *p* < 0.01) increased risk of elevated baPWV in contrast to those not presenting either OSA or high hsCRP levels. In the subgroup analysis, we also observed that the OSA participants with a high hsCRP level in the group without antihypertensive medications had a 2.37-fold (Model 1, 95% CI, 1.48, 3.79, *p* < 0.01), 1.92-fold (Model 2, 95% CI, 1.21, 3.04, *p* < 0.01), and 2.12-fold (Model 3, 95% CI, 1.33, 3.37, *p* < 0.01) elevated risk of increased baPWV in contrast to those not presenting either OSA or high hsCRP levels. However, these findings were not significant in the group with antihypertensive medications.

### 3.3. The Estimated Risk of Elevated ΔbaPWV in Relation to the Concurrent Existence of OSA and High hsCRP in Participants at 6-Year Follow-Up

The means of the baPWV and ΔbaPWV at the 6-year follow-up in terms of the existence of OSA and high hsCRP levels are presented in Table 3. The mean baPWV value of each of the four groups at the 6-year follow-up was significantly different in the subjects not treated with antihypertensive medication (n = 1348, OSA[−]/HsCRP[−] vs. OSA[−]/HsCRP[+] vs. OSA[+]/HsCRP[−] vs. OSA[+]/HsCRP[+], 13.8 ± 2.0 vs. 14.5 ± 2.1 vs. 14.7 ± 2.1 vs. 15.1 ± 2.6 m/s for baPWV, *p* < 0.01), but this was not significant in patients treated with antihypertensive medication. The multivariate analysis with adjusting for the potential confounding factors, including the obesity-associated variables and their alterations, showed that the group with both OSA and high hsCRP levels had a 1.51-fold (Model 1, 95% CI, 1.05, 2.15, *p* < 0.01), 1.48-fold (Model 2, 95% CI, 1.03, 2.12, *p* < 0.05), and 1.47-fold (Model 3, 95% CI, 1.02, 2.12, *p* < 0.05) increase in the risk of elevated ΔbaPWV in contrast to those with neither OSA nor high hsCRP levels in the entire cohort. In the subgroup analysis, we also found that the OSA participants with a high hsCRP level in the group without antihypertensive medications had a 1.68-fold (Model 1, 95% CI, 1.08, 2.61, *p* < 0.01), 1.66-fold (Model 2, 95% CI, 1.08, 2.56, *p* < 0.01), and 1.67-fold (Model 3, 95% CI, 1.08, 2.57, *p* < 0.01) increase in the risk of elevated ΔbaPWV compared to those with neither OSA nor high hsCRP levels in the group not treated with antihypertensive medication.

### 3.4. The Estimated Risk of Elevated baPWV Based on the Coexistence of MSOSA and High hsCRP

**Figure 1:**
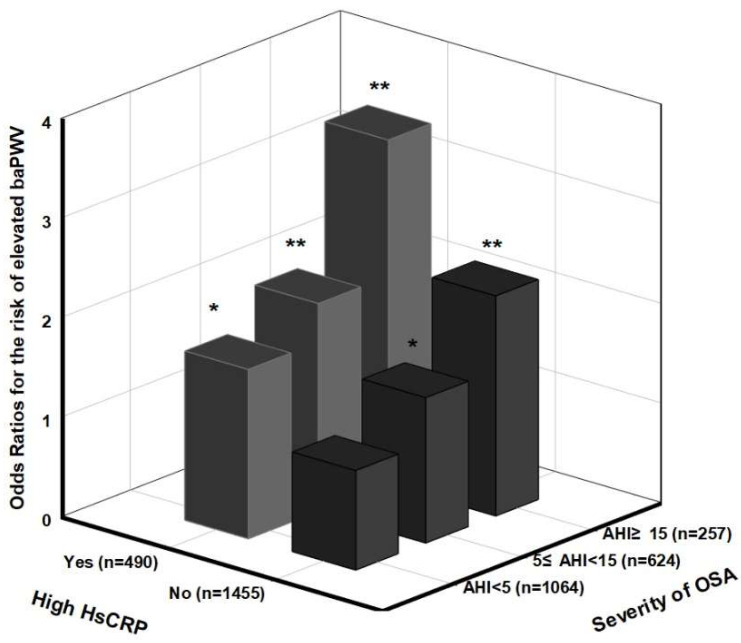
Examined odds ratios for the risk of elevated baPWV in terms of the combined condition of OSA severity and high hsCRP level in subjects not taking antihypertensive medication. Odds ratios were estimated after adjusting for age, sex, smoking, alcohol status, MAP, fasting glucose, HDL cholesterol, DM medication, hyperlipidemia medication, and BMI (n = 1945). *p*-value for interaction = 0.881. * *p* < 0.05, ** *p* < 0.01.

**Figure 2:**
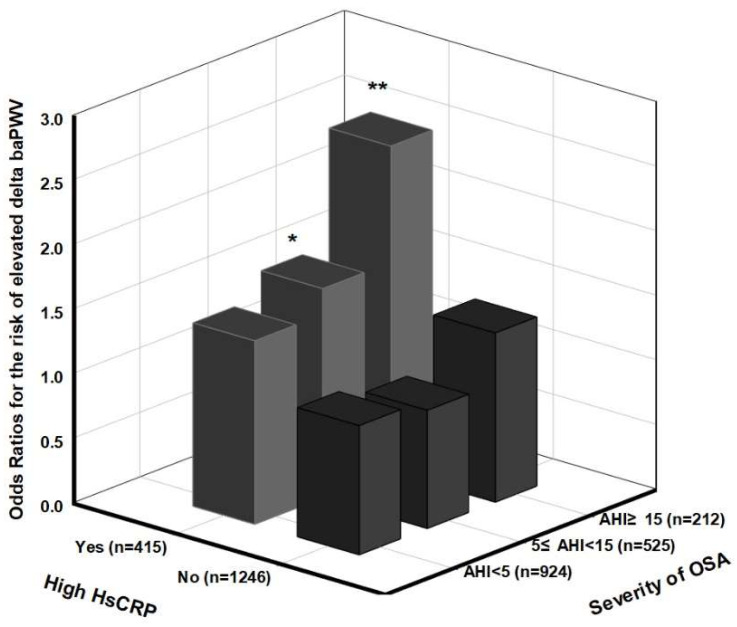
Examined odds ratios for the risk of elevated ΔbaPWV in terms of the combined condition of OSA severity and high hsCRP level in subjects with untreated hypertension at the 6-year follow-up. The odds ratio was estimated after adjusting for age, sex, smoking, alcohol status, MAP, fasting glucose, HDL cholesterol, DM medication, hyperlipidemia medication, BMI at baseline and BMI alteration (ΔBMI) at 6-year follow-up (n = 1348). *p*-value for interaction = 0.928. * *p* < 0.05, ** *p* < 0.01.

Figure 1 shows the odds ratios examined for the likelihood of the elevated baPWV according to the combined conditions of OSA severity and high hsCRP levels in subjects not treated with antihypertensive medication. In the multivariate analysis with adjustments for the potential confounding factors, including BMI, the estimated odds ratios for the elevated baPWV among the mild OSA (5 ≤ AHI < 15) and MSOSA (AHI ≥ 15) groups with high hsCRP levels were 2.09 (95% CI, 1.23, 3.55; *p* < 0.01) and 3.46 (95% CI, 1.65, 7.28; *p* < 0.01), respectively, in contrast to the non-OSA subjects with low hsCRP levels. The interaction between the OSA severity and the high hsCRP level tertile was estimated for their effects on the risk of the elevated baPWV after the adjustment for the confounding factors at the baseline. No significant differences were identified (*p*-value for interaction = 0.881). Moreover, the adjusted odds ratios for the risk of the elevated ΔbaPWV at the 6-year follow-up among the subjects divided by similar cut-off points of OSA and hsCRP are presented in Figure 2. It showed that the MSOSA subjects with high hsCRP levels had a 2.52-fold increased risk of elevated ΔbaPWV (95% CI, 1.27, 5.00; *p* < 0.01) in contrast to the non-OSA subjects with low hsCRP levels after various factors, as described above. No significant interaction between the presence of a high hsCRP level and OSA severity on the elevation of ΔbaPWV was examined (*p*-value for interaction = 0.92).

### 3.5. Alteration in baPWV in Terms of the Presence of MSOSA and High hsCRP over 6-Years Examined by the Mixed-Effects Model

**Figure 3:**
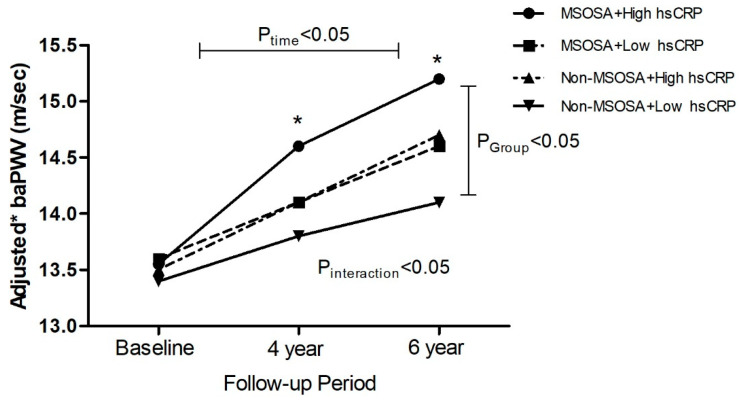
Alteration in mean baPWV after a 6-year follow-up period, according to the combined presence of high hsCRP levels and MSOSA in subjects not taking antihypertensive medications (n = 1348). *p*-values were resulted from multivariate mixed-effect linear regression models after adjustment for age, sex, smoking, alcohol status, mean arterial pressure, BMI, fasting glucose, HDL cholesterol, DM medication, hyperlipidemia medication at baseline, and alteration in BMI at 6-year follow-up. P_time_ and P_group_ present the effect of time on alteration in the baPWV over 6 years and the effect of four groups on baPWV across time, respectively; P_interaction_ presents significance of if the alteration in baPWV over time differs by four groups. * Adjusted mean baPWV in MSOSA is significantly different in contrast to that of non MSOSA and low hsCRP levels by pairwise comparisons.

Figure 3 presents the alteration in the adjusted mean baPWV over 6 years among the four groups divided by the presence of MSOSA and high hsCRP levels in participants not treated with antihypertensive medication, adjusted for confounders. Interestingly, the *p*-value, resulting from the mixed-effects linear regression analyses, showed that the adjusted mean baPWV increased with time (P_time_ < 0.05) and the groups classified by the existence of MSOSA and a high hsCRP level demonstrated an increased baPWV across time (P_group_ < 0.05). In addition, the degree of alteration in the adjusted mean baPWV over the 6-year period significantly differed based on the existence of MSOSA and high hsCRP levels (P_interaction_ < 0.05). In pairwise comparisons, the adjusted mean baPWV in the participants with the coexistence of MSOSA and a high hsCRP level was significantly higher than those without MSOSA or high hsCRP at the 4- and 6-year follow-ups.

## 4. Discussion

The concurrent existence of MSOSA and high hsCRP levels was not only related to an increased baPWV but was also significantly associated with the elevation of the ΔbaPWV. In addition, we found that the group with OSA and a high hsCRP level in the participants not treated with antihypertensive medications had a higher risk of elevated baPWV and ΔbaPWV compared to those with neither OSA nor high hsCRP levels. However, these findings were not significant in participants treated with antihypertensive medications. Interestingly, examining the mean change in the baPWV over time in terms of the presence of moderate-to-severe OSA and high hsCRP levels in the participants not treated with antihypertensive medication after adjusting for various confounding factors in a linear mixed model, we observed that the alteration in the adjusted mean baPWV over time showed a significant difference in the existence of MSOSA and high hsCRP levels (Figure 1). The adjusted mean baPWV in the group with MSOSA and high hsCRP levels was significantly increased in contrast to the groups without these variables at the 4- and 6-year follow-ups as well as at baseline, suggesting that the activated inflammatory cascades in MSOSA may be involved in accelerating the progressive arterial stiffening in subjects not taking any antihypertensive medications. To the best of our knowledge, this study is the first to examine the combined effects of OSA and inflammation regarding the progression of arterial stiffness in a prospective study. Therefore, the modifiable effect of the increase in arterial stiffening by these conditions on the development of CVD should be the focus of future studies.

So far, from the increasing body of evidence concerning OSA, it is clearly evident that OSA should be considered as a systemic inflammatory disease [5,28]. Even though the precise mechanisms implicating OSA in the inflammatory pathway have not been clearly elucidated, the systemic inflammation by repetitive hypoxia and reoxygenation characterized in OSA has appeared as a key factor in the occurrence and magnitude of OSA-related morbidity [4,29]. Not surprisingly, atherosclerosis is a disease caused by the chronic inflammatory interplays of macrophages in the artery wall, and as a result, various inflammatory biomarkers in the blood are recognized as indices for the CVD prediction [30]. The HsCRP has been used as a reliable marker of systemic inflammation and has been suggested to be a significant predictor in cardiometabolic diseases [1,31]. Substantial data from clinical and epidemiological studies have previously revealed that increased hsCRP levels are present in subjects with OSA [32,33,34] and that these levels decrease following OSA treatment [1,35,36,37]. Nonetheless, it is important to point out that not all studies have shown this presumed association between OSA and the hsCRP level [38,39], suggesting that the causal relationship between an increased CRP and OSA might not always be shown. The interaction between the severity of OSA and genetically determined variability or other environmental factors may provide an explanation for these discrepancies [1,40,41].

Arterial stiffening has increasingly been considered as a subclinical predictor associated with cardiovascular events. The most reliable vascular changes associated with increased arterial stiffening were vascular fibrosis owing to collagen deposition and considerable vessel wall calcification [11,42,43,44]. Among the various parameters for evaluating arterial stiffness, the PWV has been extensively considered as an arterial stiffening index [45,46]. Though the pathophysiological mechanisms through which the combined OSA and inflammation might increase the progressive arterial stiffening have not been clearly elucidated, accumulating evidence has shown a significant association between OSA severity and arterial stiffening in clinical and population-based studies [14,26,47,48]. Moreover, recent evidence from our group revealed that MSOSA is implicated in progressive arterial stiffening independently of obesity status at the 6-year follow-up in subjects without hypertension [15]. The CRP has not only been recognized as an indicator of systemic inflammation but it has also been associated with predictors of arterial function in several studies [30]. To date, no prospective data have examined a causal relationship by showing the acceleration of progressive arterial stiffening using the PWV among subjects presenting both OSA and inflammation. As mentioned above, both OSA and inflammation share a lot of pathophysiological pathways inflicting damage on the cardiometabolic system [49]; it is thus necessary that extensive studies attempt to examine a potential modifying effect on the progressive arterial stiffening in these conditions [50].

To examine whether the concurrent existence of OSA and inflammation may have been responsible for an exacerbated risk of progression of arterial stiffness independent of obesity-related parameters, we used three different types of variables (BMI, WHR, and FM/body weight) separately in the multiple regression models. In the present study, if we defined obesity as BMI > 25 kg/m^2^ (WHO Asia Pacific criteria), the prevalence of obesity was significantly increased according to the severity of OSA (MSOSA vs. MOSA. Non-OSA, 62.3% vs. 49.7% vs. 30.7%, *p* < 0.01). Interestingly, we found that the OSA participants presenting a high inflammatory phenotype in those not treated with antihypertensive medications had a 2.37-fold risk of elevated baPWV at the baseline (Table 2, Model 1) and a 1.68-fold increased risk of elevated ΔbaPWV at the 6-year follow-up periods (Table 3, Model 1) after adjusting for various confounders, in comparison with the corresponding non-OSA subjects with low inflammation. However, this significant association was absent in the patients treated with antihypertensive medications. Even though the exact reasons for these findings are not clearly elucidated, we suggest that antihypertensive treatment reacted in several pathways involved in hypervolemia, the sympathetic system, and the renin angiotensin system may affect the pathological mechanisms associated with the potential cardiovascular effect of OSA and the inflammatory phenotype. Previous studies have revealed that some antihypertensive medications, such as diuretics, may decrease hypervolemia and significantly reduce rostral fluid displacement during the night [51,52]. This hypervolemic condition is probably predisposed to an increase in the collapsibility of the upper airway during sleep [51]. Moreover, the result from the meta-analysis revealed that angiotensin-converting enzyme inhibitors (ACEIs) effectively reduce the PWV independent of the ability to reduce blood pressure, suggesting that treatment with ACEIs could potentially be essential to decrease arterial stiffness and the related mortality [53]. Thus, it may be speculated that antihypertensive medications might influence the progressive arterial stiffening in individuals with OSA and inflammation, as previous studies have demonstrated that the blood pressure in response to the treatment of hypertension is heterogeneous in subjects with hypertension [13]. Moreover, OSA is tightly associated with hypertension, particularly in subjects with resistant hypertension [54], indicating that untreated OSA may be attributed to the observed variability in the control of blood pressure and the end-organ damage induced by antihypertensive therapy [14]. Interestingly, a recent study has reported that no significant difference was observed in the reduction in 24-h blood pressure and arterial stiffening in hypertensive subjects with and without OSA who had antihypertensive treatment in a follow-up study [27]. Therefore, further studies are warranted to investigate the significance of antihypertensive medication on the progressive arterial stiffening in relation to OSA and inflammation in a cohort study.

Though the pathophysiological mechanisms by which the combined OSA and inflammation might accelerate the progressive arterial stiffening are not distinctly understood, activated inflammation and reactive oxygen species (ROS) have been proposed to play critical roles [13,55]. In recent decades, accumulating evidence showed that the repetitive hypoxia and reoxygenation that characterize OSA contribute to the cumulative load of oxidative stress and to the ROS generation, and these are of particular importance to processes the mediating collagen deposition and extensively vascular wall calcification [55]. Moreover, the deteriorative function of endothelial cells by increased inflammation due to OSA is involved with a marked reduction in the nitric oxide bioavailability in endothelial cells which affects the injured endothelial vasoreactivity [13,14]. It has been demonstrated that various markers for systemic inflammation, including IL-6 and TNF-α, are implicated with indices of arterial stiffening [14]. Thus, an evaluation of the combined effects of OSA and inflammatory markers or other predictors on the exacerbated risk of arterial stiffening should be examined in other studies.

This study had several limitations that need to be acknowledged. First, the evaluation of arterial stiffening applying to the baPWV among those with OSA and inflammation may inadequately represent the arterial stiffening of the central artery. Even though the carotid–femoral PWV is recognized as the gold standard for assessing the stiffening of central arteries and a biomarker for CVD, the accumulating evidence has recently revealed that the baPWV exhibits a reliable ability to expect future cardiovascular events [11,43,56], suggesting that it may be more easily applied to different settings of study than the cfPWV owing to several advantages [11]. Second, an evaluation of the inflammatory state using only the hsCRP level in patients presenting OSA may insufficiently represent the phenotypical inflammation of subjects, as other studies have suggested that other markers related to the inflammatory phenotype are implicated in OSA. Nevertheless, incremental evidence has shown that the hsCRP is a reliable inflammatory marker of the OSA-related end-organ damage and subsequent outcomes [57]. Third, the elevation of the baPWV or inflammatory conditions may be partially affected by a subject’s exercise status or nutritional habits. Several studies have suggested that aerobic exercise may improve arterial stiffening and that the effect is increased with a higher aerobic exercise intensity [58]. Accordingly, future longitudinal studies focusing on the potential modifying effects of inflammation and physical activity on the progression of arterial stiffness in individuals with OSA are needed. Finally, we did not look into the reversible causality, that is, whether OSA treatment, anti-inflammatory therapy, or a combination of the two may decrease or delay progressive arterial stiffening, thereby reducing the risk of any OSA-related consequences. Our substantial data showed that only eight subjects were receiving any OSA treatment, such as CPAP or surgical treatments, suggesting that this was not much affected in the current findings. Several studies have reported that CPAP treatment in OSA patients is significantly implicated with a decrease in arterial stiffening [12,14]. Moreover, prospective studies in patients with inflammatory disease have reported a significant improvement in the PWV after treatment with TNF-α antagonists, either alone or in combination with other agents [59,60]. Thus, more definitive studies are needed to address whether this combined treatment effectively reduces the progressive arterial stiffening.

## 5. Conclusions

In summary, individuals with moderate-to-severe OSA and subclinical inflammation not treated with antihypertensive medications had a more increased risk of the progression of arterial stiffening. These conditions are associated with an accelerated risk of a future burden of the progression of arterial stiffness, suggesting a potential important role in the increased risk of CVD.

## Figures and Tables

**Table 1:** General characteristics of study subjects in terms of the presence of OSA and inflammation. ^1)^.

	Non-OSA		OSA		*p*-Value
	HsCRP (−)	HsCRP (+)	HsCRP (−)	HsCRP (+)	
Sample size, n (%)	824 (42.4)	240 (12.3)	631 (32.4)	250 (12.9)	-
Age (years)	52.6 ± 6.3	53.9 ± 7.0 ^&^	55.9 ± 7.4	56.1 ± 7.5 ^‡^	<0.01
Male, n (%)	371 (45.0)	107 (44.6)	388 (61.5)	148 (59.2)	<0.01
BMI (kg/m^2^)	23.6 ± 2.5	24.6 ± 2.7 ^&^	24.9 ± 2.7 ^†^	26.2 ± 3.1 ^‡,§^	<0.01
ΔBMI (kg/m^2^) *	−0.01 ± 1.1	−0.09 ± 1.1	0.01 ± 1.2	−0.14 ± 1.3	0.31
WHR (cm/cm)	0.84 ± 0.06	0.86 ± 0.06 ^&^	0.87 ± 0.06 ^†^	0.89 ± 0.05 ^‡,§^	<0.01
ΔWHR (cm) *	0.039 ± 0.04	0.038 ± 0.04	0.038 ± 0.04	0.032 ± 0.03	0.054
FM/Body weight (kg/kg)	0.25 ± 0.6	0.28 ± 0.06 ^&^	0.26 ± 0.06	0.28 ± 0.07 ^‡,§^	<0.01
ΔFM/Body weight (kg/kg) *	0.02 ± 0.03	0.01 ± 0.03	0.02 ± 0.03	0.01 ± 0.03	0.053
ESS	5.9 ± 4.3	5.6 ± 4.3	5.8 ± 4.4	5.8 ± 3.8	0.75
Hypertension, n (%)	155 (18.8)	59 (24.6)	204 (32.3)	98 (39.2)	<0.01
Diabetes mellitus, n (%)	49 (5.9)	27 (11.3)	73 (11.6)	41 (16.4)	<0.01
Systolic BP at baseline (mmHg)	109.0 ± 14.1	110.9 ± 13.8	113.4 ± 14.1 ^†^	113.9 ± 13.9 ^‡,§^	<0.01
Diastolic BP at baseline (mmHg)	73.5 ± 9.8	74.8 ± 9.4	76.1 ± 9.6 ^†^	76.4 ± 9.9 ^‡,§^	<0.01
Systolic BP at follow-up (mmHg) *	113.2 ± 13.8	114.9 ± 13.0	116.6 ± 12.9 ^†^	119.2 ± 15.0 ^‡,§^	<0.01
Diastolic BP at follow-up (mmHg) *	73.4 ± 9.2	74.5 ± 8.7	74.8 ± 9.5 ^†^	75.9 ± 9.9 ^‡,§^	<0.01
AHI (events/hour)	1.9 ± 1.4	1.9 ± 1.4	12.9 ± 9.1 ^†^	15.2 ± 11.8 ^‡,§^	<0.01
(Median, IQR)	(1.70, 0.7–3.1)	(1.8, 0.6–3.1)	(9.9, 6.7–15.6)	(11.2, 7.2–17.9)	
SaO2 Nadir (%)	89.8 ± 4.8	89.9 ± 4.4	84.4 ± 4.5 ^†^	83.5 ± 5.8 ^‡,§^	<0.01
(Median, IQR)	(91.0, 89.0–92.0)	(86.0, 83.0–88.0)	(86.0, 83.0–88.0)	(82.0, 79.0–85.0)	
ODI (events/hour)	1.7 ± 1.3	1.7 ± 1.4	11.6 ± 8.5 ^†^	14.3 ± 11.7 ^‡,§^	<0.01
(Median, IQR)	(1.4, 0.6–2.8)	(1.4, 0.6–2.97)	(9.0, 6.0–14.2)	(10.1, 6.6–17.0)	
HsCRP (mg/dL) at baseline (Log transformed)	0.47 ± 0.2 (−0.41 ± 0.3)	2.59 ± 1.5 ^&^ (0.35 ± 0.2)	0.60 ± 0.3 ^†^ (−0.29 ± 0.2)	3.00 ± 1.8 ^‡,§^ (0.41 ± 0.2)	<0.01
HsCRP at follow-up (mg/dL) * (Log transformed)	0.88 ± 1.4 (−0.25 ± 0.3)	1.89 ± 2.6 (0.04 ± 0.4)	1.06 ± 1.6 ^†^ (−0.17 ± 0.3)	1.85 ± 1.8 ^‡,§^ (0.11 ± 0.3)	<0.01

BMI, body mass index; WHR, waist-to-hip ratio; FM, fat mass; ESS, Epworth Sleepiness Scale; BP, blood pressure; AHI, apnea–hypopnea index; IQR, interquartile range; ODI, oxygen desaturation index; HDL, high-density lipoprotein. ^1)^ Variables were summarized with mean ± SD. * A total of 1661 subjects were included in the analysis. ^†^ *p* < 0.01, OSA with low hsCRP vs. non-OSA with low hsCRP. ^‡^ *p* < 0.01, OSA with high hsCRP vs. non-OSA with low hsCRP. ^&^ *p* < 0.05, non-OSA with high hsCRP vs. non-OSA with low hsCRP. ^§^ *p* < 0.05, OSA with high hsCRP vs. non-OSA with high hsCRP.

**Table 2:** The mean value of baPWV and odds ratios for the risk of increased baPWV in terms of the presence of OSA and inflammatory status in subjects with or without antihypertensive medication at baseline.

		Odds Ratios for the Risk of Elevated baPWV (95% CI) ^1)^				
		OSA (−)/ HsCRP(−)	OSA (−)/ HsCRP(+)	OSA (+)/ HsCRP(−)	OSA (+)/ HsCRP(+)	*p*-Value ^†^
With HTN medication (n = 396)	Sample size, n (%)	105 (26.5)	44 (11.1)	164 (41.4)	83 (21.0)	-
	MAP (mmHg)	88.8 ± 8.7	90.1 ± 8.6	91.5 ± 9.3	91.5 ± 10.5	>0.05
	BaPWV (m/s)	14.6 ± 2.5	14.6 ± 2.1	14.4 ± 2.0	15.0 ± 2.4	>0.05
	Elevated baPWV n, (%) ^1)^	42 (40.0)	18 (40.9)	60 (36.6)	41 (49.4)	>0.05
	Unadjusted	Reference	1.03 (0.50–2.12)	0.86 (0.52–1.43)	1.46 (0.81–2.61)	>0.05
	Adjusted Model 1	Reference	0.96 (0.43–2.14)	0.61 (0.33–1.11)	1.24 (0.61–2.52)	>0.05
	Adjusted Model 2	Reference	0.94 (0.41–2.12)	0.56 (0.31–1.02)	1.09 (0.55–2.17)	>0.05
	Adjusted Model 3	Reference	0.96 (0.42–2.18)	0.58 (0.31–1.06)	1.16 (0.57–2.33)	>0.05
Without HTN Medication (n = 1549)	Sample size, n (%)	719 (46.4)	196 (12.7)	467 (30.1)	167 (10.8)	-
	MAP (mmHg)	84.8 ± 10.8	86.1 ± 10.5	87.5 ± 10.5	87.7 ± 10.4	<0.01
	BaPWV (m/s)	13.1 ± 1.8	13.6 ± 2.1	13.8 ± 1.9	14.1 ± 1.9	<0.01
	Elevated baPWV n, (%) ^1)^	104 (14.5)	46 (23.5)	123 (26.3)	54 (32.3)	<0.01
	Unadjusted	Reference	1.81 (1.22–2.67) ^&^	2.11 (1.57–2.83) ^&^	2.82 (1.92–4.15) ^&,^*	<0.01
	Adjusted Model 1	Reference	1.70 (1.06–2.67) ^&^	1.62 (1.14–2.31) ^&^	2.37 (1.48–3.79) ^&,^*	<0.01
	Adjusted Model 2	Reference	1.53 (1.01–2.42) ^§^	1.43 (1.01–2.02) ^§^	1.92 (1.21–3.04) ^&,^*	<0.01
	Adjusted Model 3	Reference	1.63 (1.03–2.59) ^§^	1.51 (1.06–2.14) ^§^	2.12 (1.33–3.37) ^&,^*	<0.01
All (n = 1945)	Sample size, n (%)	824 (42.4)	240 (12.7)	631 (12.3)	250 (12.9)	-
	MAP (mmHg)	85.3 ± 10.7	86.8 ± 10.3	88.5 ± 10.3	88.9 ± 10.6	<0.01
	BaPWV (m/s)	13.3 ± 1.9	13.8 ± 2.1	14.0 ± 1.9	14.4 ± 2.2	<0.01
	Elevated baPWV n, (%) ^1)^	146 (17.7)	64 (26.7)	183 (29.0)	95 (38.0)	<0.01
	Unadjusted	Reference	1.89 (1.48–2.43) ^&^	1.68 (1.20–2.36) ^&^	2.84 (2.08–3.88) ^&,^*	<0.01
	Adjusted Model 1	Reference	1.50 (1.01–2.23) ^§^	1.24 (0.92–1.68)	2.05 (1.39–3.01) ^&,^*	<0.01
	Adjusted Model 2	Reference	1.38 (0.93-2.05)	1.11 (0.83–1.50)	1.67 (1.15–2.43) ^&,^*	<0.01
	Adjusted Model 3	Reference	1.46 (0.98–2.17)	1.17 (0.87–1.57)	1.84 (1.26–2.69) ^&,^*	<0.01

MAP, mean arterial pressure; BaPWV, brachial–ankle pulse wave velocity. ^§^ *p* < 0.05, ^&^ *p* < 0.01, ^†^ the combinatory association is significant for *p*-value < 0.05. * *p* < 0.05, OSA(−)/HsCRP(+) vs. OSA (+)/HsCRP(+). ^1)^ Elevation of baPWV determined as greater than the cut-off of highest quartile level. Adjusted Model 1: age, sex, smoking, alcohol status, MAP, fasting glucose, HDL cholesterol, DM medication, hyperlipidemia medication, and BMI. Adjusted Model 2: age, sex, smoking, alcohol status, MAP, fasting glucose, HDL cholesterol, DM medication, hyperlipidemia medication, and WHR. Adjusted Model 3: age, sex, smoking, alcohol status, MAP, fasting glucose, HDL cholesterol, DM medication, hyperlipidemia medication, and FM/body weight.

**Table 3:** The mean ΔbaPWV and odds ratios for the risk of elevated ΔbaPWV in relation to the presence of OSA and inflammatory status in subjects with or without antihypertensive medication at the 6-year follow-up.

		Odds Ratios for the Risk of Elevated baPWV (95% CI) ^1)^				
		OSA (−)/ HsCRP(−)	OSA (−)/ HsCRP(+)	OSA (+)/ HsCRP(−)	OSA (+)/ HsCRP(+)	*p*-Value ^†^
With HTN medication (n = 313)	Sample size, n (%)	85 (27.2)	38 (12.1)	128 (40.9)	62 (19.8)	-
	BaPWV (m/s)	15.6 ± 2.9	16.1 ± 2.9	15.9 ± 2.7	15.8 ± 3.0	>0.05
	ΔBaPWV (m/s)	1.19 ± 2.3	1.47 ± 2.5	1.46 ± 2.2	1.27 ± 1.9	>0.05
	Elevated ΔbaPWV n, (%) ^1)^	26 (30.6)	13 (34.2)	47 (36.7)	19 (30.6)	>0.05
	Unadjusted	Reference	1.18 (0.52–2.66)	1.31 (0.73–2.36)	1.00 (0.49–2.04)	>0.05
	Adjusted Model 1	Reference	1.07 (0.45–2.51)	1.24 (0.65–2.37)	0.92 (0.42–2.04)	>0.05
	Adjusted Model 2	Reference	1.02 (0.43–2.39)	1.13 (0.60–2.12)	0.84 (0.39–1.82)	>0.05
	Adjusted Model 3	Reference	1.06 (0.45–2.53)	1.18 (0.62–2.24)	0.86 (0.39–1.91)	>0.05
Without HTN medication (n = 1348)	Sample size, n (%)	630 (46.7)	171 (12.7)	404 (30.0)	143 (10.6)	-
	BaPWV (m/s) ^†^	13.8 ± 2.0	14.5 ± 2.1	14.7 ± 2.4	15.1 ± 2.6	<0.01
	ΔBaPWV (m/s)	0.78 ± 1.4	0.97 ± 1.7	0.89 ± 1.8	1.06 ± 2.0	>0.05
	Elevated ΔbaPWV n, (%) ^1),†^	122 (19.4)	50 (29.2)	91 (22.5)	48 (33.6)	<0.01
	Unadjusted	Reference	1.72 (1.17–2.52) ^&^	1.21 (0.89–1.64) ^&^	2.10 (1.41–3.13) ^&^	<0.01
	Adjusted Model 1	Reference	1.62 (1.08–2.41) ^&^	0.99 (0.71–1.38)	1.68 (1.08–2.61) ^&^	<0.01
	Adjusted Model 2	Reference	1.59 (1.07–2.37) ^§^	1.00 (0.72–1.39)	1.66 (1.08–2.56) ^&^	<0.01
	Adjusted Model 3	Reference	1.57 (1.05–2.36) ^§^	0.98 (0.70–1.36)	1.67 (1.08–2.57) ^&^	<0.01
All (n = 1661)	Sample size, n (%)	715 (43.0)	209 (12.6)	532 (32.0)	205 (12.4)	-
	BaPWV (m/s) ^†^	14.0 ± 2.2	14.8 ± 2.3	15.0 ± 2.5	15.3 ± 2.7	<0.01
	ΔBaPWV (m/s)	0.82 ± 1.6	1.06 ± 1.8	1.02 ± 1.9	1.13 ± 2.0	0.068
	Elevated ΔbaPWV n, (%) ^1),†^	148 (20.7)	63 (30.1)	138 (25.9)	67 (32.7)	<0.01
	Unadjusted	Reference	1.65 (1.16–2.33) ^&^	1.34 (1.02–1.74) ^§^	1.86 (1.31–2.62) ^&,^**	<0.01
	Adjusted Model 1	Reference	1.46 (1.02–2.13) ^&^	1.07 (0.80–1.44)	1.51 (1.05–2.15) ^&,^**	<0.05
	Adjusted Model 2	Reference	1.43 (0.98–2.08)	1.08 (0.82–1.44)	1.48 (1.03–2.12) ^§,^**	<0.05
	Adjusted Model 3	Reference	1.43 (0.98–2.08)	1.07 (0.80–1.43)	1.47 (1.02–2.12) ^§,^**	<0.05

^§^*p* < 0.05, ^&^ *p* < 0.01, ^†^ the combinatory association is significant for *p*-value < 0.05 (*p* < 0.01), ** *p* < 0.05, OSA(+)/HsCRP(−) vs. OSA(+)/HsCRP(+). ^1)^ Elevation of ΔbaPWV determined as greater than the cut-off of highest tertile level of ΔbaPWV. Adjusted Model 1: age, sex, smoking, alcohol status, MAP, BMI, fasting glucose, HDL cholesterol, DM medication, hyperlipidemia medication at baseline, and alteration in BMI (ΔBMI) at 6-year follow-up. Adjusted Model 2: age, sex, smoking, alcohol status, MAP, WHR, fasting glucose, HDL cholesterol, DM medication, hyperlipidemia medication at baseline, and alteration in WHR (ΔWHR) at the 6-year follow-up. Adjusted Model 3: age, sex, smoking, alcohol status, MAP, FM/body weight, fasting glucose, HDL cholesterol, DM medication, hyperlipidemia medication at baseline, and alteration in FM/body weight (ΔFM/body weight) at 6-year follow-up.

## Data Availability

The data underlying this article will be shared on reasonable request to the corresponding author.
